# The implementation of omics technologies in cancer microbiome research

**DOI:** 10.3332/ecancer.2018.864

**Published:** 2018-09-05

**Authors:** Benjamin H Mullish, Laura S Osborne, Julian R Marchesi, Julie AK McDonald

**Affiliations:** 1 Division of Integrative Systems Medicine and Digestive Disease, Department of Surgery and Cancer, Faculty of Medicine, Imperial College London, London SW7 2AZ, UK; 2 Microbiomes, Microbes and Informatics Research Department, School of Biosciences, Cardiff University, Cardiff CF10 3AT, UK

**Keywords:** microbiome, metataxonomics, metagenomics, metabonomics, metaproteomics

## Abstract

Whilst the interplay between host genetics and the environment plays a pivotal role in the aetiopathogenesis of cancer, there are other key contributors of importance as well. One such factor of central and growing interest is the contribution of the microbiota to cancer. Even though the field is only a few years old, investigation of the ‘cancer microbiome’ has already led to major advances in knowledge of the basic biology of cancer risk and progression, opened novel avenues for biomarkers and diagnostics, and given a better understanding of mechanisms underlying response to therapy. Recent developments in microbial DNA sequencing techniques (and the bioinformatics required for analysis of these datasets) have allowed much more in-depth profiling of the structure of microbial communities than was previously possible. However, for more complete assessment of the functional implications of microbial changes, there is a growing recognition of the importance of the integration of microbial profiling with other omics modalities, with metabonomics (metabolite profiling) and proteomics (protein profiling) both gaining particular recent attention. In this review, we give an overview of some of the key scientific techniques being used to unravel the role of the cancer microbiome. We have aimed to highlight practical aspects related to sample collection and preparation, choice of the modality of analysis, and examples of where different omics technologies have been complementary to each other to highlight the significance of the cancer microbiome.

## Introduction

The past few years have been associated with a surge of interest in the potential contribution of the microbiota to a number of human diseases, including a wide range of cancers [1]. The streamlining of protocols for the optimal collection and processing of samples from mucosal sites—coupled with the rapid refinement of culture-independent techniques for profiling the microbiota from these samples—has meant that microbial profiling has become an area of focus for clinicians and scientists engaged in cancer research. One central recurring question in cancer microbiome research is regarding the mechanisms of interaction between the microbiota and the host, and the role of specific metabolites and proteins appears to be key [1]. As such, in addition to microbial profiling, the use of other omics technologies—in particular, metabonomics and proteomics—contributes highly to understanding the functional implications of microbiome changes. In this review, we will give a summary of some popular omics technologies available to researchers as they apply to cancer microbiome research (metataxonomics, metagenomics, metabonomics and metaproteomics).

## Study design and sample types

There are a variety of variables that have been shown to influence the composition and/or functionality of the gut microbiota, including (but not limited to) diet, medications (especially antibiotics), age, surgery, stress, chemotherapeutic drugs, probiotics, body mass index, pregnancy and microbial infections [2–4]. Detailed clinical data must be recorded for each study participant to identify these confounding variables, and these variables must be considered when matching participants in case–control studies. Due to the large impact that antibiotics have on the gut microbiota, many studies exclude participants which have used antibiotics within the previous 3–6 months. Moreover, the microbiota present in both faecal and biopsy samples is affected by bowel preparation procedures performed prior to colonoscopies. Shobar *et al* [5] found short-term changes in the composition of the microbiota following bowel preparation; however, changes affected diversity metrics differently in mucosal and faecal samples, and in healthy and inflammatory bowel disease (IBD) samples.

The most common sample type used in human gut microbiome studies is faecal samples. Faecal samples are noninvasive and can be collected by participants at home using commercial kits. Although there are significant differences in the composition of faecal samples and mucosal biopsy samples [6], faecal samples are widely accepted as a more practical alternative to biopsy samples for diagnostic purposes. Indeed, studies have shown differences in the composition of the faecal microbiota of colorectal cancer patients and healthy participants, with studies consistently reporting enrichment of *Peptostreptococcus stomatis*, *Parvimonas micra*, *Porphyromonas* spp. and *Fusobacterium nucleatum* in the faecal microbiota of colorectal cancer patients [6–10].

The gut mucosal microbiota are thought to be particularly important to host health due to the proximity of microbial cells to the host epithelium. Therefore, biopsy samples are thought to provide more meaningful mechanistic data to studies investigating host–microbe interactions compared to faecal samples. Mucosal microbiota samples are often taken through pinch biopsy during colonoscopy or during bowel surgery, and therefore characterisation of the mucosal microbiota could also be useful as a diagnostic tool [11]. Comparison of biopsies from colorectal cancer patients and healthy controls can be difficult, as it is not ethical to ask healthy volunteers to undergo a colonoscopy to collect healthy mucosal biopsy samples. However, some studies recruit healthy volunteers undergoing colonoscopies for colon cancer screening [12]. Other options for sampling the mucosal microbiota include luminal brushing (superficial sampling of tissues) [13, 14], submucosal sampling (deeper sampling of tissues) [15] or colonic lavage samples [12]. Previous studies have explored changes in the mucosal microbiota in patients with colorectal cancer. Nakatsu *et al* [16] found that *Fusobacterium*, *Parvimonas*, *Gemella* and *Leptotrichia* were most significantly enriched in early-stage colorectal cancer. In another study, Kinross *et al* [17] demonstrated that *Fusobacteria* and ε-*Proteobacteria* were enriched on tumour tissue compared to adjacent normal mucosal tissue, and the relative abundance of *Fusobacteria* and β-*Proteobacteria* increased with advancing cancer stage.

## DNA extraction

We recommend extracting DNA from samples within 3 months of storage at −80°C to avoid potential changes in the composition of the stool sample over time [18]. It is important to include a mechanical lysis step (bead beating) in the DNA extraction protocol to ensure proper cell lysis of Gram-positive bacteria and archaea [19, 20]. Bead beating is performed by mixing faecal samples with 0.1-mm glass or silica beads and buffer and homogenising with a bead beating instrument. There are many options for the DNA extraction protocol used; however, researchers often choose to use commercial kits. Following both the mechanical and chemical lysis using these kits, the DNA is purified by mixing the cell lysate with a series of buffers, binding of the DNA to an immobilised matrix (e.g. a column, magnetic beads), washing the bound DNA, and eluting the purified DNA.

Unfortunately, the DNA extraction protocol used for gut microbiota studies is not standardised, and there are a wide variety of DNA extraction kits used in published studies [21]. It is important to use the same DNA extraction protocol for all samples to be compared for DNA sequencing studies, as differences in the DNA extraction kits results in differences in the DNA yield and profile of the microbial community [22, 23]. However, regardless of the DNA extraction protocol chosen for a study, the microbial DNA extracted will still be subject to some degree of bias, e.g. some bacterial cells are easier to lyse than others.

It can be challenging to obtain adequate DNA concentrations from small biopsy samples. In metataxonomic studies, sequencing of low biomass samples results in sequencing data containing contaminant bacterial DNA sequences originating from DNA extraction kit reagents [24, 25]. We advise researchers sequencing low biomass samples to also sequence several DNA kit controls where the DNA extraction protocol is carried out in the absence of a sample. In metagenomic studies, low bacterial biomass biopsy samples result in the undesired sequencing of human DNA. Therefore, we do not advise researchers to perform metagenomic analysis on these samples as it is very difficult to obtain adequate sequencing coverage of bacterial DNA, unless the researcher is prepared to pay for the sequencing data.

## Metataxonomic sequencing (16S rRNA gene sequencing)

Metataxonomic sequencing is a technique where researchers determine the bacterial composition of a sample by polymerase chain reaction (PCR) amplifying and sequencing one or more variable regions of the 16S rRNA gene present in bacterial genomes from the extracted DNA sample. The 16S rRNA gene is present in all prokaryotes, and consists of nine hypervariable regions of differing sequences and length, interspaced by highly conserved regions [26, 27]. Primers are designed to bind to conserved regions of the gene that flank one or more of the hypervariable regions [28]. Because the DNA sequences of these hypervariable regions are phylogenetically distinct for a given species, sequencing of these regions allows researchers to determine the bacterial composition of each sample. Metataxonomic sequencing can reliably classify bacterial taxa down to the genus level in human samples (and in some taxonomic cases, species level), and down to the family level in murine samples [29].

Following amplification and purification of one or more variable regions of 16S rRNA gene, the PCR product for each sample is used as a template in a second PCR reaction that adds a unique combination of barcoded indices to each sample [30]. These barcoded indices allow many samples to be pooled and sequenced in parallel on a single flow cell (up to 384 samples for one sequencing run). The amplicons are purified, mixed together at equimolar concentrations, denatured and sequenced. Illumina and ThermoFisher produce several sequencing instruments, with the Illumina MiSeq and the ThermoFisher Ion Personal Genome Machine being the most popular for metataxonomic sequencing. These instruments are capable of sequencing 300–400 bp paired-end reads [31, 32].

A disadvantage of metataxonomic sequencing is that different bacteria contain different copy numbers of the 16S rRNA gene (2–15 copies per bacterial genome) [33], which can bias the composition of the sample by making some bacteria look more abundant than they actually are in the sample. The composition of the sample also depends on how well the universal primers can amplify a bacterial species, and therefore this method is susceptible to PCR bias [34]. Moreover, the choice of hypervariable regions, primer sequences and PCR conditions are not standardised, and there can be significant differences in the sequencing results obtained from the same sample [34, 35]. Therefore, researchers are advised to use the same primers and PCR conditions for all samples from the same study and are cautioned when comparing data between studies where samples were prepared following different protocols.

## Shotgun metagenomic sequencing

Shotgun metagenomic sequencing is a technique where researchers fragment and randomly sequence DNA from the collection of genomes and genes present in the extracted DNA sample. In addition to gaining taxonomic data from a sample, characterisation of the metagenome allows researchers to gain additional information including the functional potential of the microbiota. This is because shotgun metagenomics sequences most of the genes present in the sample, in contrast to metataxonomic sequencing which only targets part of the 16S rRNA gene. Moreover, shotgun metagenomic sequencing can more reliably classify bacteria down to species/strain level compared to metataxonomic sequencing [36].

Shotgun metagenomic sequencing also requires a larger number of sequencing reads to provide adequate sequencing coverage of all the genes in the sample. Therefore, researchers often use the Illumina HiSeq to perform these sequencing runs. Following sequencing, the DNA sequences of these fragments are assembled or mapped to a reference database and annotated, or a database-independent approach may be used [37].

However, there are several challenges associated with shotgun metagenomic sequencing. Annotation of genes from data sets is dependent on the accuracy and completion of reference databases and genomes. Therefore, metagenomic data sets can have a large proportion of reads corresponding to genes with unknown functions, and in some cases, this can be as high as 60% of the total reads [38]. In contrast to metataxonomic sequencing (which PCR amplifies a region of the 16S rRNA gene), shotgun metagenomic sequencing shears the genomic DNA in the sample, and therefore is not subject to PCR bias at this step. However, shotgun metagenomic sequencing is still subject to biases due to the DNA extraction step, index PCR step, sequencing errors and so on. Moreover, while shotgun metagenomic sequencing can provide users with more information than metataxonomic sequencing, the sequencing process itself is significantly more expensive (shotgun metagenomic sequencing is generally 20–30 times more expensive than metataxonomic sequencing) [35]. It is also challenging to process, store and analyse the large data sets resulting from shotgun metagenomic sequencing.

Although it is unarguable that metataxonomic and shotgun metagenomic sequencing have helped to achieve significant advances in the field of cancer regarding diagnosis, prognosis and treatment, there are intrinsic limitations associated with these techniques that do not allow us to perceive the complete picture [39]. Shotgun metagenomic sequencing helps us to identify genes that might be involved in cancer, but it is not clear if these genes are being actively expressed. Another method to assess whether a given microbial gene is being actively expressed is to use metatranscriptomics, where complementary DNA (cDNA) is synthesised from transcribed microbial genes (mRNA) and sequenced. However, this technique is more challenging, complex and expensive, especially for intestinal biopsy samples where microbial mRNA is present at much lower levels compared to mRNA levels from host tissue, and with respect to the significant abundance of cDNA derived from rRNA, which can be the major component of an analysis if it is not removed. To understand gut microbiota function and interactions in their entirety in a given pathological state other techniques are required, such as metabonomics and metaproteomics.

## Metabonomics

Metabonomics may be defined as ‘the quantitative measurement of the dynamic multi-parametric metabolic response of multicellular systems to pathophysiological stimuli’ [40]. More specifically, it enables the detection, identification and quantification of metabolites responsible for mediating the phenotypic expression of altered metabolism resulting from a biological challenge. Metabolites that are identified may represent products of host metabolism, microbial metabolism or co-metabolism between microbiota and host. Metabonomics, therefore, provides a valuable tool in elucidating potential mechanistic links between alterations in microbial composition and changes to host physiology, including the development of the disease. Metabonomics is conducted by analysing biofluids using powerful spectroscopic techniques—in particular, nuclear magnetic resonance (NMR) spectroscopy and/or mass spectrometry (MS)—with the subsequent use of advanced multivariate statistical tools to interrogate the spectral data produced.

The metabolite profile obtained from biofluids is highly sensitive to factors that may vary in sample collection, including the length of time between the collection of sample and preparation for spectroscopy, or the temperature at which the sample has been collected and stored. As such, standardised protocols for sample collection and preparation for metabolite analysis have been designed and optimised for a range of biofluids, including plasma, serum, urine, tissue extracts [41], stool/faecal supernatant [42] and breath [43]. It is even possible to metabolically analyse intact tissue using a technique called ‘magic angle spinning’ [44]. A wide range of different forms of spectroscopic analysis are available (targeted versus untargeted assays, negative versus positive mode and so on; Table 1), with the most suitable assays dependent upon the metabolites of interest, biofluids that are available, and cost amongst other factors. In many studies comparing biofluids from those with cancer to those of matched healthy participants, one strategy taken has been to perform a global metabolite screen with a technique such as NMR first, identify possible metabolite groups of interests that differentiate samples, and then to perform a targeted technique (often MS) to explore these metabolites in greater detail [45]. The raw spectral data that is generated is complex and multi-stage analysis under expert supervision is required, including the separation of signal from noise, alignment of peaks, normalisation of data and the use of both unsupervised and supervised statistical techniques to allow full interpretation [46]. Other complexities lie in understanding how the metabonomic data obtained link to the biology of the system under investigation—for example, do metabolites that change after an intervention reflect modifications in the host, microbial modifications or alterations in host-microbial crosstalk?

There have been a variety of applications of metabonomics as a means to better understand the contribution of the microbiome to disease pathogenesis or outcomes in cancer. The gradual emergence of studies co-reporting microbiome and metabolite profiles on the same sample set is the first step towards progress in understanding this relationship [17]; further studies applying this same approach—but using longitudinal sampling, and/or samples across a range of tumour progression (from pre-malignant through to metastatic malignant states)—promise further elucidation of relevant mechanisms.

One future avenue of interest is likely to be an expansion of ‘pharmacomicrobiomics’ [47], i.e. using metabonomics as a contributory tool to better understand the interactions between the metabolic and immune functions that the microbiota exert on a host with or at risk of cancer, and the drugs used to treat it. If successful, this could allow a new paradigm for all levels of cancer care, where manipulation of microbiota metabolic functionality could be applied to slow progression of cancer, optimise selection of or response to systemic treatment and so on [47].

## Exoproteomics and oncoproteomics

Exoproteomics is a technique that studies the protein content found in the extracellular proximity of a given biological system, e.g. the luminal content of the gut. The emerging use of exoproteomics in a clinical cancer setting (or ‘oncoproteomics’) enables the identification of specific proteins, abundances, structures, interactions and post-translational modifications in a given biological state. There is a growing body of evidence demonstrating the utility of proteomics in cancer research, including the identification of biomarkers for early diagnosis. One key example includes the first Food and Drug Administration approved protein biomarker, OVA1, discovered following proteome profiling from sera samples, and now used for the detection of early-stage ovarian cancer [48].

Oncoproteomic methodologies used to discover such biomarkers are becoming increasingly powerful and robust, including the bioinformatics pipelines required for data interpretation. The major methods involved include gel-based and gel-free analysis, MS and microarray-based methods [49]. While detailed descriptions of the methodologies are beyond the scope of this article, they have been described in detail elsewhere [49–53]. The general workflow of a proteomics pipeline involves several steps, one of the most important of which is sample preparation. Sample preparation involves the extraction and separation of protein usually from tissue biopsies, serum and/or plasma. Sample collection, storage and processing should be conducted with consistency and under stringent standard operating procedures [49]. Separation of proteins can be achieved via gel-based analysis, namely, 2D gel electrophoresis. Gel-free methods coupled with MS—such as high-performance liquid chromatography, isotope-coded affinity tags and stable isotope labelling by amino acids in cell culture (SILAC) [39]—may also be used. Protein identification techniques using MS have become the ‘gold standard’ analytical methodologies in oncoproteomic pipelines. Following MS, the entire proteome of a particular sample can be assayed via computational analysis to give a library of peptides with protein type, abundance, functions and structure. Matrix-assisted laser desorption/ionisation time-of-flight MS (described in detail by Singhal *et al* [54]) has emerged as an efficient and accurate means for routine proteomics. The technique has assisted in the analysis and validation of several biomarkers, including those related to the identification and diagnosis of infectious diseases, as well as those related to cancer [55].

However, it is important to note that the application of proteomics to cancer microbiome research is certainly less advanced than that of DNA-based analyses. One of the main reasons for this is the complexity of the proteome. The host proteome alone consists of an estimated one to several million proteins encoded by around 20,000 genes, with differing biochemical and physical properties, often interacting as a network rather than on their own [56], and these estimations are even higher when extending the proteome to include the microbiome. Projects such as the Human Proteome project (https://hupo.org/human-proteome-project) have aimed to define the human proteome, and are making progress via a concerted effort to coordinate different research laboratories regarding sharing and exchanging the generated data and methodologies used. However, discrepancies in proteome profiles generated using varied methodologies in different research laboratories is still a major problem [57]. Furthermore, MS techniques may also introduce significant bias to a complex biological sample due to a lack of sensitivity for detecting low abundance proteins. In addition, at present, the process is also significantly lower throughput than DNA sequencing platforms, and the technology too complex for routine use in a clinical setting.

High-throughput microarray-based technologies as a complement to MS represent a means by which cancer microbiome proteomics may advance. The most developed version of these, reverse phase protein microarrays, immobilise a number of different cellular lysates or biological specimens onto the microarray and screen using a range of antibodies for the expression of proteins of interest, all within a single chip (for a detailed description of the methodologies, see Sutandy *et al* [58]).

One of the key ways that proteomic technologies can aid in cancer microbiome research is by studying the effects of the gut microbiota on the pharmacodynamics of cancer therapy drugs. The gut microbiota have been shown to be central to the bioavailability of certain anticancer drugs and the efficacy of certain treatments, particularly immunotherapy [59, 60]. In a study by Stringer *et al* [61], a combination of traditional proteomics and metatransciptomics was utilised to characterise dysbiosis of the gut microbiota during chemotherapy. They found that chemotherapy-induced diarrhoea could be attributed to alterations in the gut microbiota, resulting in increased levels of several proteins which could act as toxicity biomarkers for patients at risk of developing chemotherapy-induced diarrhoea, including faecal calprotectin, circulating matrix metalloproteases, NF-ҡβ, IL-1β and TNF.

Proteomic techniques can also provide valuable information for cancer research *in vitro*. Kim *et al* [62] implemented MS proteome profiling of *Escherichia coli* and *Staphylococcus aureus* culture supernatants to screen for outer membrane vesicles (OMVs). OMVs are extracellular vesicles often produced by Gram-negative bacteria with lipid-bilayers containing numerous immunostimulatory factors. They demonstrated that trypsin-sensitive surface proteins of these extracellular vesicles were capable of inducing anticancer cytokines (interferon-γ and CXCL10) in a mouse model of colon adenocarcinoma, resulting in a significant reduction in tumour burden [63].

Bacterial oncoproteomics holds the potential to not only accelerate diagnosis and increase treatment efficacy but also to identify mechanisms of cancer prevention by disrupting certain key microbial pathways [64]. For oncoproteomics to reach its full potential in a clinical setting, however, there is a global need for the standardisation of sample collection, preparation and bioinformatic processing.

## Example of the integrated application of omic technologies to cancer microbiome research

A relevant application of omics technologies to cancer research is the study of the microbial contribution to the toxicity of cancer chemotherapeutics. For example, Lin *et al* [65] explored the mechanisms by which CPT-11 (also known as irinotecan, a chemotherapeutic used to treat colorectal malignancy) has a common side effect of chronic diarrhoea. Initial studies showed CPT-11 increased the relative abundances of *Clostridium* cluster XI and *Enterobacteriaceae* in the caecal content of tumour-bearing rats. Studies also showed that chronic diarrhoea can be minimised after co-administration of dietary fibre with CPT-11 [66]. However, the reduction in CPT-11 toxicity with dietary fibre did not correlate with stimulation of specific bacterial taxa, although there was a close correlation with caecal concentrations of the short chain fatty acid butyrate, and the bacterial butyryl CoA gene [66]. Proteomic studies have also shown that β-glucuronidases produced by the gut microbiota are responsible for reactivating CPT-11, which plays a role in diarrhoea and can prevent dose intensification and efficacy [67]. Such toxic side effects can be ameliorated with the use of inhibitors targeting these bacterial β-glucuronidases. These and other similar studies have emphasised that rather than just the presence or absence of specific bacterial taxa as a predictor of chemotherapeutic toxicity, the metabolic functionality of the microbiota may be more important.

## Conclusion

Whilst there have been several interesting studies investigating the role of the gut microbiota in cancer pathogenesis, there are still many questions that need to be answered. For example, while the application of omic technologies can show differences in the mucosal microbiota of colorectal cancer patients, it is not clear whether these bacteria are the cause of cancer initiation, or whether a developing tumour creates an environmental niche that favours the growth of opportunistic bacteria. Omic technologies are a powerful tool to identify bacteria, metabolites or proteins that correlate with cancer presence or cancer stage progression. However, carefully controlled animal studies or longitudinal studies in humans are required to confirm the role of these identified targets in cancer pathogenesis.

## Authors’ contributions

BHM, LSO and JAKM wrote the initial draft of the manuscript and received editorial input from JRM. All authors read and edited the final version of the manuscript.

## Conflicts of interest

Nil for all authors.

## Figures and Tables

**Table 1.** Summary of common spectroscopic assays that may be of use to cancer microbiome research.

Technique	Specific form	Notes
NMR		Most of use for broad profiling of a range of metabolites Higher reproducibility than MS.
MS	Gas chromatography	This is particularly useful for the identification and quantification of volatile metabolites such as short-chain fatty acids
	Liquid chromatography	Can be used in ‘profiling’ (i.e. detection, identification and relative quantification of a particular group of metabolites, e.g. bile acids, lipids, etc), or as a ‘targeted’ technique (i.e. specific identification and accurate quantification of each member of a group of metabolites, e.g. individual amino acids) The instrument can be used in positive or negative ionisation mode, depending upon the specific metabolites of interest May be performed with chromatography step performed in a number of different ways, including Hydrophilic interaction chromatography: Useful for identifying small polar molecules Reverse phase chromatography: Detects a broad range of metabolites, ranging from moderately polar to moderately apolar molecules
	Desorption electrospray ionisation (DESI)	A form of ambient MS, where intact samples, in their native environment, are directly ionised. The instrument used to perform ionisation is directly linked to a mass spectrometer
	Rapid evaporative ionisation MS (REIMS)	A further form of ambulatory MS. A radiofrequency electrical current is applied to a sample via a stainless steel monopolar probe. The vapour generated contains gas phase ions of metabolites and structural lipids. The vapour is channelled to a mass spectrometer attached to the instrument using an incorporated vacuum system
